# Dietary Patterns, Kidney Function, and Sarcopenia in Chronic Kidney Disease

**DOI:** 10.3390/nu17030404

**Published:** 2025-01-23

**Authors:** Ji Woo Kim, Soo Jin Yang

**Affiliations:** Department of Food and Nutrition, Seoul Women’s University, Seoul 01797, Republic of Korea

**Keywords:** chronic kidney disease, dietary patterns, protein energy wasting, sarcopenia

## Abstract

Sarcopenia is a condition characterized by the loss of muscle mass and function. It is a risk factor for adverse clinical outcomes, including falls, disability, and mortality in patients with chronic kidney disease (CKD). The progression of CKD leads to metabolic disturbances and pathophysiological changes. These alterations, such as metabolic acidosis, dysregulated muscle proteostasis, and excessive inflammation, contribute to accelerated muscle wasting, resulting in sarcopenia. Proper nutritional interventions are essential in the management of sarcopenia in patients with CKD. Appropriate dietary intake of protein and specific micronutrients, carefully considering the needs and restrictions of CKD, may help maintain muscle mass and function. Specific dietary patterns, such as an anti-inflammatory diet, Dietary Approaches to Stop Hypertension diet, and a plant-based diet, may be beneficial for attenuating muscle wasting in CKD patients. The underlying mechanisms of how these dietary patterns affect sarcopenia are multifaceted, including inflammation, oxidative stress, and defects in muscle protein homeostasis. This review summarizes the current evidence on the relationship between dietary patterns and sarcopenia, as well as the underlying mechanisms of how dietary patterns modulate sarcopenia in CKD patients.

## 1. Introduction

Sarcopenia is a muscle disorder characterized by a decline in muscle mass and function [1]. Multiple organizations and expert groups have proposed different versions of definitions and criteria for diagnosing sarcopenia, reflecting its multifaceted etiologies and pathophysiology [2,3,4,5,6]. Although these consensus guidelines principally agree with the basic concept that sarcopenia is a condition characterized by the loss of muscle mass and function, they differ in the diagnostic components and cut-off values of measures related to muscle health. Recent global collaborative efforts were initiated to reach an agreement regarding the definition of sarcopenia by the Global Leadership Initiative in Sarcopenia group [7,8].

Sarcopenia increases the risk for adverse clinical outcomes, including falls, hip fracture, disability, and mortality [9], and its prevalence is significant in chronic diseases, such as chronic kidney disease (CKD) [10]. The recent global systematic review and meta-analysis estimated 24.5% as the global prevalence of sarcopenia in CKD patients by pooling a total of 140 studies across 25 countries [10]. Age, physical inactivity, disease-specific pathophysiological changes (e.g., hypercatabolism, uremic toxins, and inflammation), and protein-energy wasting (PEW)/malnutrition may contribute to the increased risk for sarcopenia in CKD [11,12,13].

Nutritional interventions for sarcopenia include adequate intake of energy, protein, amino acids (e.g., leucine), vitamin D, and omega-3 polyunsaturated fatty acids (PUFAs) in the general population [14,15]. Application of these interventions for CKD requires caution, considering the needs and restrictions associated with CKD. Moreover, given that PEW and sarcopenia result from overall malnutrition rather than deficiency of specific nutrients, dietary patterns as a practical nutritional strategy for these conditions need to be considered for the management of CKD. Although accumulating evidence is limited, an anti-inflammatory diet, Dietary Approaches to Stop Hypertension (DASH) diet, and a plant-based diet have been suggested as potential dietary interventions to prevent or improve PEW and sarcopenia in kidney diseases [16,17,18]. Mechanisms of developing sarcopenia include inflammation, oxidative stress, defects in muscle protein homeostasis [19,20], and the modulation of these mechanisms may be potential ways underlying how specific dietary patterns attenuate muscle wasting and dysfunction in CKD.

This review aims to summarize the current evidence on the relationship between specific dietary patterns and sarcopenia, as well as the underlying mechanisms through which these dietary patterns attenuate muscle wasting in CKD. This review, therefore, provides information for developing dietary guidelines aimed at mitigating muscle loss and dysfunction in CKD patients.

## 2. Sarcopenia in CKD

Sarcopenia is prevalent among patients with CKD, and its occurrence increases as CKD advances [10,11]. The prevalence of sarcopenia and severe sarcopenia in CKD is relatively high, accounting for 24.5% and 21%, respectively, having a higher prevalence for patients on dialysis [10]. Among the three components (low muscle strength, low muscle mass, and low physical performance) for diagnosing sarcopenia, low muscle strength had the highest rate of 43.4% in CKD [10].

**Figure 1:**
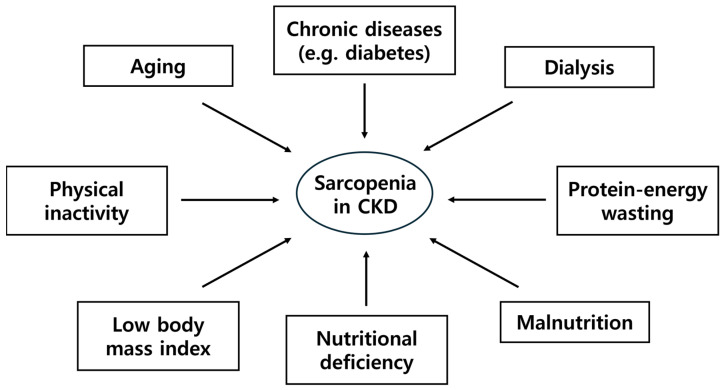
Risk factors for sarcopenia in chronic kidney disease (CKD).

Risk factors for sarcopenia in CKD include aging, chronic diseases such as diabetes, dialysis, PEW, malnutrition, nutritional deficiency, low body mass index, and reduced physical activity [21] (Figure 1). Patients with CKD frequently experience PEW and malnutrition due to dietary restrictions, reduced appetite, and metabolic disturbances [13]. Additionally, patients with CKD may experience difficulties with chewing due to dental issues, and changes in taste perception as aging advances, leading to reduced appetite and dietary intake [22]. In addition, nutritional deficiency of protein, essential amino acids, and vitamins (e.g., vitamin D) can exacerbate muscle loss [14,15].

The recent clinical practice guideline from the Kidney Disease: Improving Global Outcomes suggests maintaining a protein intake of 0.8 g/kg body weight/day, not exceeding more than 1.3 g/kg body weight/day in adults with CKD [23]. The nutritional strategy to maintain protein intake of 0.8 g/kg body weight/day or limit protein intake below of 0.8 g/kg body weight/day is beneficial for preventing or delaying decline in kidney function [24]. A low-protein diet (≤0.6 g protein/kg body weight/day) during the 4–6 months follow-up period does not affect muscle mass and function [25]. However, a long-term low-protein diet is often accompanied by reduced dietary intake and/or energy deficiency, causing protein-energy malnutrition and PEW conditions, subsequently developing sarcopenia [26]. Current evidence suggests that protein intake of 0.8 g/kg body weight/day would be appropriate for CKD patients, especially when sarcopenia is present, or in elderly patients with CKD [27].

Sarcopenia is more prevalent in patients on dialysis compared to non-dialysis [10]. Muscle wasting becomes severe due to a combination of reduced food intake, metabolic disturbance, and inflammation in patients on dialysis. Dialysis itself can exacerbate muscle loss due to inflammation, the catabolic process of nutritional metabolism, and the loss of nutrients including protein and amino acids during dialysis [28]. Therefore, nutritional management to delay the development and progression of sarcopenia needs to consider the stages of CKD and whether the patient is currently undergoing dialysis. Certain dietary patterns may be applicable only for non-dialysis patients, and traditional nutritional approaches may be more appropriate for patients on dialysis. In addition to dietary management, exercise helps improve muscle mass and function. Recently, intra-dialytic exercise was emphasized for patients with end-stage kidney disease as a practical strategy to delay the development and progression of sarcopenia [29,30].

Kidney transplantation is expected to improve sarcopenia in CKD. The pooled prevalence of sarcopenia from a meta-analysis on kidney transplantation recipients was 26% [31]. Previous analyses reported that the prevalence of sarcopenia varies from 4% to 72% in kidney transplantation recipients [32,33]. According to a comparative analysis of patients with CKD, sarcopenia was observed in 5% in non-dialysis-dependent CKD patients, 12% in hemodialysis, 9% in peritoneal dialysis, and 4% in kidney transplant recipients [32]. These large variations in the prevalence of sarcopenia among kidney transplant recipients are possibly due to the differences in diagnostic criteria of sarcopenia and sample sizes. Therefore, it is not clear whether kidney transplantation can improve sarcopenia in CKD based on the current evidence.

Sarcopenia in CKD is associated with higher mortality rates [34]. The loss of muscle mass and function reduces physical strength, making patients more vulnerable to complications like infections, falls, and fractures [6]. Muscle wasting leads to decreased physical strength and increased risk of frailty and adverse health clinical outcomes.

## 3. Pathophysiology and Mechanisms of Sarcopenia in CKD

**Figure 2:**
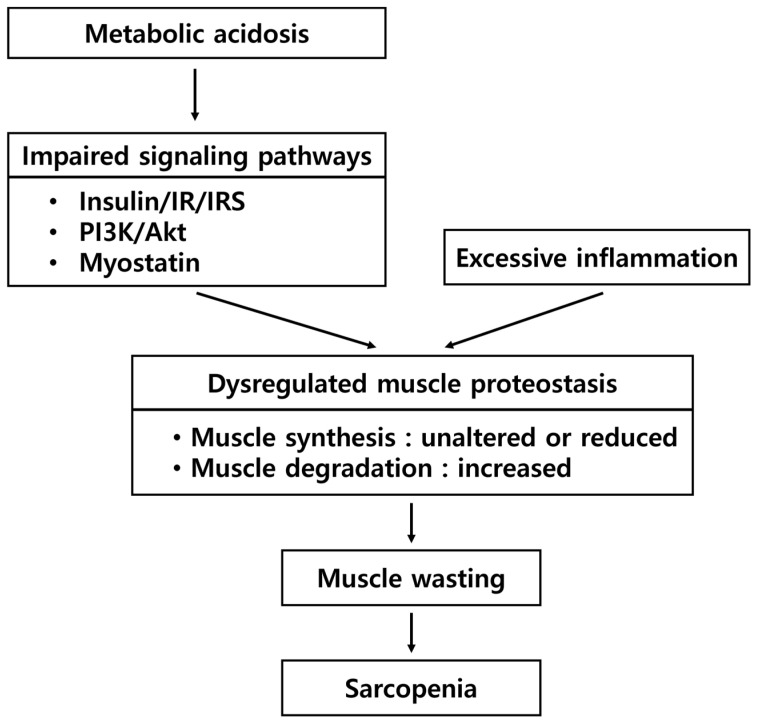
Pathophysiological changes and mechanisms of sarcopenia in chronic kidney disease. IR—insulin receptor; IRS—IR substrate; PI3K—phosphatidylinositol 3-kinase.

As CKD progresses, pathophysiological changes in metabolism follow [35]. Muscle wasting is a major characteristic of CKD-related sarcopenia and is driven by multiple molecular mechanisms related to dysregulated muscle proteostasis, insulin resistance, metabolic acidosis, and excessive muscle inflammation [36,37,38,39] (Figure 2).

### 3.1. Muscle Wasting

Muscle wasting is a condition in which muscle breakdown exceeds muscle synthesis [40], and is accelerated with PEW, malnutrition, inflammation, and metabolic acidosis. Decreased kidney function was linked to muscle wasting and reduction in muscle mass, which results in sarcopenia [41,42]. Muscle wasting and sarcopenia are associated with mortality in patients with CKD [34]. Insufficient intake of protein and energy or imbalanced nutritional intake resulted in malnutrition, which is a main risk factor for muscle loss and muscle wasting contributing to the onset of sarcopenia [43]. Traditional dietary regimen for CKD management often limits dietary intake, leading to PEW and malnutrition and subsequently accelerating muscle wasting [35].

### 3.2. Dysregulated Muscle Proteostasis

In CKD, the balance between protein synthesis and degradation is disrupted, leading to a loss of muscle mass [44]. Dysregulated muscle protein homeostasis is considered the most relevant mechanism for muscle wasting. The anabolic pathway of muscle protein was unaffected or reduced, but the catabolic pathway was activated in CKD-related sarcopenia [45].

This imbalance is driven by various factors relating to the uremic milieu.

As a major risk factor for CKD, diabetes affects approximately half of diabetic patients and ranks among the primary cause of end-stage kidney disease [46,47]. Insulin is a hormone that favors synthetic metabolic processes [48]. Impaired insulin action or low concentrations of insulin in insulin resistance or diabetes resulted in abnormalities in protein metabolism, reducing muscle protein synthesis [48]. A higher prevalence of sarcopenia was reported in patients with diabetes, confirming that insulin dysfunction was related to sarcopenia [49]. Although no conclusive studies exist, the prevalence of sarcopenia is likely higher in CKD patients with concurrent diabetes compared to those without diabetes.

Metabolic acidosis is a condition that accumulates endogenous acids and is diagnosed when bicarbonate levels fall below 22 mEq/L [50]. The prevalence of metabolic acidosis is estimated to be 20% in CKD patients and increases as CKD progresses [50,51]. In an acidotic rodent model, metabolic acidosis increased skeletal muscle breakdown by the activation of ubiquitin-proteasome-dependent pathway [52] and glucocorticoid-mediated action [53]. In addition, metabolic acidosis condition was associated with declines in gait performance and physical function in individuals with and without CKD [54,55]. Metabolic acidosis stimulated protein degradation by interfering with the action of insulin in patients with CKD [56]. Another study on long-term hemodialysis patients altered blood bicarbonate concentrations using bicarbonate supplements and demonstrated that acidosis accelerated muscle breakdown, which was corrected by bicarbonate supplements [57]. The catabolic effects of metabolic acidosis on skeletal muscle are mediated, in part, by impaired insulin signaling involving the insulin/insulin receptor (IR)/IR substrate pathway and phosphatidylinositol 3-kinase/Akt pathway. Subsequently, metabolic acidosis-mediated impairment of insulin signaling activates the ubiquitin-proteasome system and caspase-3, enhancing protein catabolism [58]. Another catabolic factor relating to metabolic acidosis is the myostatin signaling pathway. Myostatin is a protein that inhibits muscle growth, and it is often elevated in CKD patients [59]. In addition to metabolic acidosis, excessive inflammation and oxidative stress also increase myostatin synthesis [59].

### 3.3. Inflammation

Inflammation is associated with the pathophysiological alterations of CKD [38,60]. The pro-inflammatory marker, C-reactive protein (CRP), was elevated in CKD patients [61,62] and was negatively correlated with the estimated glomerular filtration rate and albumin [62]. Also, the circulating levels of other pro-inflammatory cytokines, interleukin-6 (IL-6), and tumor necrosis factor (TNF)-alpha were higher in patients with CKD [63]. Moreover, a higher systemic immune–inflammation index, estimated by neutrophil, lymphocyte, and platelet counts, increased the odds for CKD [64]. Another systemic inflammation-related index, the systemic inflammation response index, calculated by neutrophil, monocyte, and lymphocyte counts, was also associated with CKD [64]. These two peripheral systemic inflammation-related indices were associated with mortality in CKD [64,65].

Sarcopenia can develop not only from factors unrelated to dialysis but also from inflammation caused by the dialysis process itself [10]. Dialysis treatment results in the loss of nutrients, including protein, during dialysis and can promote inflammation through multiple mechanisms. It can cause infections and immune system activation from central line catheters, involves contact with potentially incompatible dialysis membranes, and may provide inadequate waste removal. In peritoneal dialysis, specifically, additional inflammatory triggers include infections of the peritoneum and catheter tunnel, ongoing exposure to dialysis fluid, and stress from advanced glycation end products building up in the body [66,67].

Excessive inflammation is suggested to accelerate muscle degradation [35]; however, direct evidence linking inflammation and muscle degradation in CKD is limited. It has been hypothesized that increased inflammation in CKD induces muscle protein degradation, which is accompanied by excessive levels of pro-inflammatory cytokines, nuclear factor-kappa B, CRP, IL-6, and Janus kinase/signal transducer and activator of the transcription pathway. These alterations lead to muscle cell senescence and reduced synthesis of muscle proteins, possibly interacting with Akt, AMP-activated protein kinase, and mitogen-activated protein kinase pathways [35].

## 4. Dietary Patterns and Their Impact on Sarcopenia in CKD

Dietary patterns, the overall combinations of foods consumed regularly, reflecting habitual dietary intake, play a crucial role in the management and mitigation of sarcopenia in patients with CKD. Up to this point, dietary guidelines for CKD have focused more on limiting specific nutrients instead of suggesting dietary patterns. This approach was applied due to two main challenges: the practical difficulty of implementing dietary patterns and the lack of strong evidence supporting specific dietary patterns for CKD. We reviewed the current evidence on the relationship between dietary patterns and sarcopenia in CKD. Despite limited evidence, an anti-inflammatory diet, a DASH diet, and a plant-based diet have been suggested as potential dietary interventions for CKD. While other dietary patterns, such as the Mediterranean diet and ketogenic diet, were initially considered, they were ultimately excluded due to insufficient evidence regarding their relationship with, or impact on, sarcopenia in CKD. The representative evidence regarding the relationship between dietary patterns, kidney function, and sarcopenia is summarized in Table 1.

### 4.1. Anti-Inflammatory Diet

An anti-inflammatory diet is a dietary pattern that emphasizes eating nutrient-dense foods known for reducing chronic and low-grade inflammation in the body, such as fruits, vegetables, whole grains, nuts, and legumes [68]. Chronic and low-grade inflammation is linked to a wide array of health issues, including obesity, insulin resistance, diabetes, heart diseases, certain cancers, autoimmune conditions, and neurodegenerative disorders [69,70,71,72]. Moreover, CKD is often accompanied by excessive levels of pro-inflammatory cytokines CRP, IL-6, and TNF-alpha [61,62,63], which may aggravate the progression of sarcopenia [35].

The principal components contributing to anti-inflammatory properties are plant-based ingredients from fruits and vegetables, healthy fats such as omega-3 PUFAs, and healthy proteins from beans, lentils, fatty fish, and lean poultry. Specifically, phytochemicals and antioxidants in plant-based ingredients combat oxidative stress and inflammation, and omega-3 PUFAs and healthy proteins from non-red meat food resources reduce inflammation [68].

An anti-inflammatory diet in CKD may be beneficial for reducing cardiovascular risk in that anti-inflammatory nutrients (e.g., omega-3 PUFAs and antioxidants) may improve endothelial function and reduce atherosclerosis progression [68,73]. In addition to the cardioprotective effects of the anti-inflammatory diet, the diet has the potential to slow CKD progression [74,75,76]. While more research is needed, lower inflammation may positively influence kidney function decline rates and reduce proteinuria, a marker of kidney damage.

**Figure 3:**
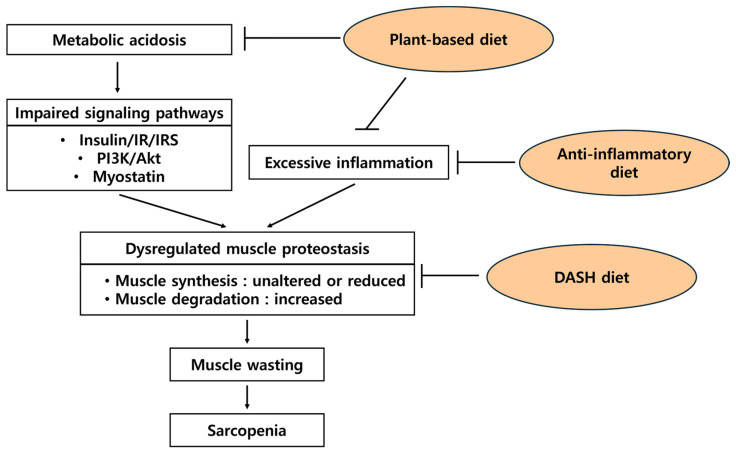
Suggested mechanisms of how dietary patterns affect sarcopenia in chronic kidney disease. IR—insulin receptor; IRS—IR substrate; PI3K—phosphatidylinositol 3-kinase.

CKD is frequently associated with chronic systemic inflammation and metabolic disturbances that contribute to muscle wasting and reduced muscle strength [21]. An anti-inflammatory diet may help mitigate these inflammatory processes, potentially preserving muscle mass and function in CKD (Figure 3). While direct clinical trials specifically testing the hypothesis that “anti-inflammatory diet prevents or improves sarcopenia in CKD” are limited, the association of dietary inflammatory potential and sarcopenia was analyzed on the public database of the National Health and Nutrition Examination Survey (NHANES) of the United States [16]. The cross-sectional study of 2569 adult CKD participants in NHANES reported that dietary inflammatory index, an index of dietary inflammatory potential, was independently related to sarcopenia and positively associated with sarcopenia in participants with CKD [16]. This evidence suggests that reducing inflammation through dietary intervention may preserve muscle mass and function, mitigating sarcopenia progression in patients with CKD.

### 4.2. DASH Diet

The DASH diet is a well-established dietary pattern developed to help lower and control high blood pressure [77]. It emphasizes foods that are naturally lower in sodium and higher in potassium, magnesium, calcium, and fiber [77]. The DASH diet recommends eating fruits, vegetables, whole grains, and lean protein sources (e.g., poultry, fish, legumes, low-fat dairy foods, and nuts), and limits sodium, saturated fat, cholesterol, and added sugar [77]. Beyond its well-known benefits for reducing blood pressure and improving cardiovascular health, the DASH diet is also linked to improved health conditions, including obesity and metabolic syndrome [78,79]. Also, higher adherence to the DASH diet is associated with improved kidney function, especially in the early stages of CKD, by reducing blood pressure and decreasing the load on the kidneys [80,81]. Lower sodium intake and moderate amounts of potassium, magnesium, and fiber in fruits and vegetables help lower blood pressure, preventing a decline in kidney function [80,81].

The progressive decline in kidney function affects nutritional metabolism and muscle mass, which can exacerbate sarcopenia [82]. Moderate lean protein contents in the DASH diet may help preserve muscle mass and function in patients with CKD. There is inconsistent evidence on the DASH diet and sarcopenia. A study on the community-dwelling older adult population did not observe any significant association between the DASH diet and sarcopenia [83]. Another cross-sectional study on autosomal dominant polycystic kidney disease reported that higher adherence to the DASH diet was negatively associated with lower handgrip strength, a component of sarcopenia [18]. In general, the DASH diet is a nutrient-dense diet with balanced micronutrients and lean protein sources, making it beneficial for preserving muscle mass and function (Figure 3).

The DASH diet can improve musculoskeletal health, but patients need careful monitoring of their protein, potassium, and phosphorus intake and blood levels to stay within the individually prescribed limits. Excess amounts of protein, potassium, and phosphorus can stress the kidneys by increasing the load to excrete nitrogenous waste, potassium, and phosphorus. If the monitoring of these nutrients is possible, the DASH diet would be a compatible dietary strategy for patients with CKD to maintain musculoskeletal health.

### 4.3. Plant-Based Diet

A plant-based diet is a dietary pattern of eating foods primarily from plants, including fruits, vegetables, legumes, whole grains, nuts, seeds, and plant-based oils, limiting animal foods [84]. A plant-based diet is considered a dietary pattern that is abundant in nutrients and has health benefits on weight management, type 2 diabetes, hypertension, and certain cancers [85,86]. Key nutrients to consider for its health benefit are fiber, plant protein, antioxidants, phytonutrients, and micronutrients [84].

Its initial approach for CKD was a low-protein plant-based diet, which may be effective in treating and slowing the progression of CKD [87,88]. Research has shown that when patients followed a vegetarian diet very low in protein and supplemented with ketoanalogues, their need for dialysis was postponed [89]. However, this treatment approach is best suited for patients with strong motivation, as they must consume a large number of ketoanalogue pills daily [90].

The benefits of a plant-based diet are relatively lower levels of acid load, uremic toxins, phosphorus, and lower risk of hyperfiltration compared with animal-based diets [84,88]. Metabolic acidosis is accompanied by the progression of CKD because the kidneys become less efficient at excreting acid [91,92]. The plant-based diet is generally lower in acid-forming nutrients and higher in base-forming fruits and vegetables, potentially improving acid–base balance [84,91]. Better control of acidosis is linked to slower progression of CKD [91,92]. Also, plant proteins tend to produce fewer nitrogenous waste products than animal proteins, and higher dietary fiber intake from the plant-based diet helps reduce the production of gut-derived uremic toxins and excrete excess acids, phosphorus, and potassium [84]. Recent analyses showed that the plant-based diet or plant protein was effective for preserving or improving kidney function, preventing the development of incident CKD and delaying the progression of CKD [93,94,95].

The benefits of the plant-based diet on kidney function are relatively well-researched. However, its effects on sarcopenia in CKD have not yet been extensively investigated. A recent study on the association between a plant-based diet and the risk of PEW and sarcopenia reported that higher adherence to the plant-based diet had a lower risk of PEW, but no association with sarcopenia in patients with CKD [17]. The plant-based diet is a way to provide moderate amounts of protein and micronutrients for musculoskeletal health to maintain muscle mass and muscle function. Also, the plant-based diet reduces acid load and has anti-inflammatory and anti-oxidative capacities [84], which may reduce excessive muscle inflammation and improve dysregulated muscle proteostasis in sarcopenic conditions (Figure 3). Concerns of the plant-based diet for patients with CKD are the risk of PEW and hyperkalemia. When well-managed, a plant-based diet helps lower the risk of PEW or prevent the occurrence of hyperkalemia [17,96].

**Table 1:** The relationship between dietary patterns, kidney function, and sarcopenia in chronic kidney disease (CKD).

Dietary Patterns	Study Design	Outcomes	Ref.
Anti-inflammatory diet	Cross-sectional study, 2569 CKD participants from National Health and Nutrition Examination Survey (NHANES)	The prevalence of sarcopenia was 19.11% of patients with CKD. The dietary inflammatory potential was positively associated with sarcopenia in patients with CKD.	[16]
Anti-inflammatory diet	Cross-sectional study, 9824 participants from the Ravansar non-communicable diseases (RaNCD)	Higher adherence to a pro-inflammatory diet was positively associated with a risk of CKD.	[97]
Anti-inflammatory diet	Cohort study, 1422 participants	Higher dietary inflammatory index (DII) score was associated with reduced renal function at baseline and a greater renal function decline over 10 years.	[98]
Anti-inflammatory diet	Cross-sectional study, 21,649 participants from NHANES	Pro-inflammatory diet was associated with kidney function decline and high risk of CKD.	[75]
Anti-inflammatory diet	Cross-sectional study, 2488 CKD participants from NHANES	Higher DII score was associated with an increased risk of CKD progression and reduced kidney function.	[76]
DASH diet	Cross-sectional study, 68 participants with autosomal dominant polycystic kidney disease	Higher adherence to DASH diet was associated with low risk of reduced handgrip strength.	[18]
DASH diet	Cross-sectional study, 1110 CKD participants from NHANES	Low adherence to a DASH diet was associated with higher risk of end-stage kidney disease in patients with CKD.	[80]
DASH diet	Cohort study, 1630 participants from the Tehran Lipid and Glucose Study	Higher adherence to a DASH diet was associated with lower risk of incident CKD.	[99]
Plant-based diet	Cohort study, 14,686 participants from Atherosclerosis Risk in Communities study	Healthy plant-based diet and a provegetarian diet were negatively associated with risk of CKD.	[94]
Plant-based diet	Cross-sectional study, 9746 participants from RaNCD	Higher adherence to a plant-based diet was associated with lower risk of CKD.	[100]
Plant-based diet	Cross-sectional study, 109 participants with kidney diseases	Plant-based diet was negatively associated with a risk of protein energy wasting, but had no significant relationship with the risk of sarcopenia	[17]

DASH, Dietary Approaches to Stop Hypertension.

## 5. Conclusions

Sarcopenia is a critical health condition often accompanied by the development and progression of CKD. Sarcopenia in CKD is derived from various nutrition-related factors, such as PEW, malnutrition, and nutritional deficiencies. Therefore, multidisciplinary approaches, including nephrologists, dietitians, physical therapists, and family caregivers, are crucial for managing sarcopenia in CKD patients effectively.

Moderate dietary intakes of protein, amino acids, and certain micronutrients are recommended for the prevention and treatment of sarcopenia. However, the application of these approaches for patients with CKD is limited because of the increased risk of decline in kidney function, hyperkalemia, and hyperphosphatemia. Therefore, dietary patterns have more attention than approaches focused on individual nutrients.

Here, the possibilities of an anti-inflammatory diet, DASH diet, and a plant-based diet as a compatible dietary regimen for patients with CKD were discussed, considering the aspects of kidney function and sarcopenia. Collectively, appropriate dietary protein intake and adequate energy intake would help prevent the development of sarcopenia in patients with CKD. Additionally, the dietary patterns reviewed here may be applicable for patients with early stages of CKD. However, close nutritional and medical supervision should be provided, and the above-mentioned dietary approaches should be applied with caution, especially for those at high risk of progressing to end-stage kidney disease and elderly CKD patients, because the existing evidence is unclear regarding the appropriate dietary intake of protein and plant foods (e.g., fruits and vegetables) when considering both kidney function and sarcopenia.
